# Adverse association of epicardial adipose tissue accumulation with cardiac function and atrioventricular coupling in postmenopausal women assessed by cardiac magnetic resonance imaging

**DOI:** 10.3389/fcvm.2022.1015983

**Published:** 2022-11-11

**Authors:** Shan Huang, Ke Shi, Li Jiang, Yan Ren, Jin Wang, Wei-Feng Yan, Wen-Lei Qian, Yuan Li, Zhi-Gang Yang

**Affiliations:** ^1^Department of Radiology, West China Hospital, Sichuan University, Chengdu, Sichuan, China; ^2^Department of Endocrinology, West China Hospital, Sichuan University, Chengdu, Sichuan, China

**Keywords:** epicardial adipose tissue, left atrial strain, left ventricular strain, cardiac magnetic resonance imaging, postmenopausal women

## Abstract

**Background:**

This study aims to investigate the association of epicardial adipose tissue (EAT) accumulation with cardiac function and atrioventricular coupling in a cohort of postmenopausal women assessed by cardiac magnetic resonance imaging (CMR).

**Materials and methods:**

Overall, 283 postmenopausal women (mean age 61.5 ± 9.1 years) who underwent CMR examination were enrolled. Participants were classified into four groups by the quartile of EAT volume. EAT volume was quantified on short-axis cine stacks covering the entire epicardium. CMR-derived cardiac structure and function, including left atrial (LA)- volume, emptying fraction, deformation, and left ventricular (LV)- mass, volume, ejection fraction, and deformation, were compared among the four groups of graded EAT volume.

**Results:**

Left ventricular mass (LVM) and LV remodeling index were both increased in the group with the highest EAT volume, compared to those in the lowest quartile (*p* = 0.016 and *p* = 0.003). The LV global longitudinal strain (LV-GLS), circumferential strain (LV-GCS), and LA- reservoir strain (LA-RS), conduit strain (LA-CS), and booster strain (LA-BS), were all progressively decreased from the lowest quartile of EAT volume to the highest (all *p* < 0.05). Multivariable linear regression analyses showed that EAT was independently associated with LV-GLS, LA-RS, LA-CS, and LA-BS after adjusting for body mass index and other clinical factors.

**Conclusion:**

Epicardial adipose tissue accumulation is independently associated with subclinical LV and LA function in postmenopausal women. These associations support the role of EAT in mediating deleterious effects on cardiac structure and function.

## Introduction

During menopausal transition, women are susceptible to metabolic alterations. Redistribution of body fat from the subcutaneous area to the intra-abdominal visceral area is an important metabolic change for women after menopause. Previous studies have shown that epicardial adipose tissue (EAT) volume tends to be expanded particularly in postmenopausal women (1, 2). EAT was indicated to be associated with hypertension, coronary microvascular dysfunction, and diastolic filling restriction in women but not in men (1, 3, 4). In addition, studies have shown that in the presence of hemodynamic stress, women tend to present more frequently with left ventricular (LV) hypertrophy, smaller LV volumes, and preserved ejection fraction, compared to age-matched men (5, 6). These notable sex differences indicate that estrogen deficiency and EAT might play a role in mediating cardiac abnormalities in postmenopausal women.

Cardiac magnetic resonance (CMR) imaging has been widely used in the evaluation of heart structure and function. CMR tissue tracking has been validated to have excellent producibility and reproducibility in evaluating LV myocardial deformation. Recently, the importance of left atrial (LA) phasic function and feature tracking strain has been increasingly recognized. A prior study indicated that LA dysfunction preceded the onset of heart failure (7). CMR-derived phasic LA function and strain have been suggested to be able to serve as sensitive imaging biomarkers in the assessment and stratification of diastolic dysfunction (8, 9). Furthermore, CMR has been validated to be able to quantify the epicardial fat tissue using a simple volumetric technique on a standard clinical steady-state free-precession sequence (10).

To the best of our knowledge, few studies on this subject have assessed EAT volume and cardiac structure and function by using CMR. Therefore, we aimed to investigate the association of EAT accumulation with cardiac function and atrioventricular coupling in a cohort of postmenopausal women by CMR.

## Materials and methods

### Study population

This study was approved by the Biomedical Research Ethics Committee of our hospital and conducted in accordance with the Declaration of Helsinki. Written informed consent was waived due to the retrospective nature of this study.

In this cross-sectional study, we included a cohort of 283 postmenopausal women who underwent CMR examination between January 2015 and June 2021. The menopausal status of the participants was recorded according to self-report. Women with both natural menopause and surgical menopause were included. The demographic and clinical characteristics of the included individuals were recorded according to digital medical records. Triglyceride-to-high density lipoprotein cholesterol ratio (TG/HDL) was calculated to indicate insulin resistance level (11).

Exclusion criteria were as follows: (a) patients with pericardial effusion; (b) atrial fibrillation; (c) obstructive coronary artery disease and myocardial infarction, (d) myocarditis and pericarditis, (e) moderate to severe valvular disease, (f) primary and secondary cardiomyopathies, and (g) poor image quality and unavailable to derive CMR parameters.

### Cardiac magnetic resonance protocol

All CMR scans were performed using a 3.0T scanner (Siemens Healthcare, Erlangen, Germany). Balanced steady-state free-precession sequence was used to obtain cine images. Three long-axis views (2-chamber, 3-chamber, 4-chamber) and consecutive short-axis slices covering the entire LV were obtained with the following parameters: temporal resolution, 33.22 ms; repetition time, 2.77 ms; echo time, 1.31 ms; field of view, 234 mm × 280 mm and slice thickness, 8 mm.

### Imaging analysis

#### Measurement of left ventricular structure and function

All CMR parameters were assessed using a commercially available software (CVI^42^; Circle Cardiovascular Imaging, Inc., Calgary, AB, Canada). LV structural parameters, including LV end-diastolic volume (LV-EDV), LV end-systolic volume (LV-ESV), and LV mass (LVM), were attained by manually tracing the endocardial and epicardial contours of the left ventricle at the end-diastolic and end-systolic phases on the short-axis stacks. LV functional parameters included LVEF and LV myocardial strain. LV myocardial strain indices were acquired by loading the short-axis stacks and the two-chamber and four-chamber long-axis images into the feature tracking module. The software then computes the LV global radial (LV-GRS), circumferential (LV-GCS), and longitudinal peak strain (LV-GLS) (12).

#### Measurement of left atrial structure and function

**FIGURE 1:**
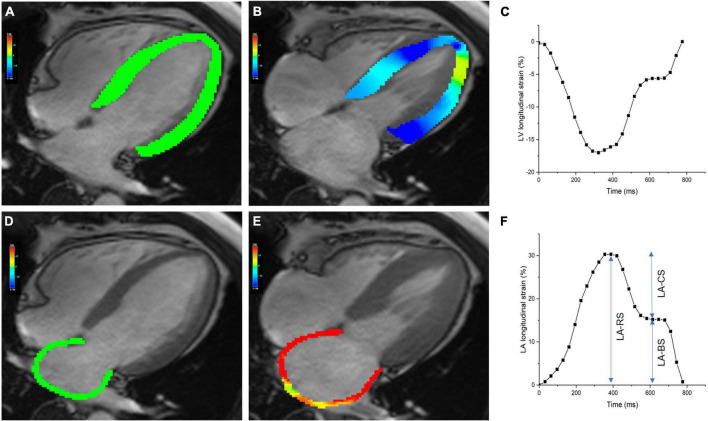
Cardiac magnetic resonance (CMR)-derived left ventricular and atrial longitudinal strain. Panels **(A,B)** show the pseudocolor maps of LV longitudinal strain in the four-chamber view at the end-diastolic and end-systolic phases. Panel **(C)** shows representative plot of LV longitudinal strain. Panels **(D,E)** show the pseudocolor maps of LA longitudinal strain in the four-chamber view at the end-diastolic and end-systolic phases. Panel **(F)** shows a plot of LA longitudinal strain, along with measures of reservoir, conduit, and booster strain. LA-RS, LA reservoir strain; LA-CS, LA conduit strain; LA-BS, LA booster strain.

Left atrial parameters were obtained as previously described (13). LA structural parameters included maximum LA volume (LAV_*max*_), minimum LA volume (LAV_*min*_), and pre-atrial contraction LA volume (LAV_*ac*_). LA functional parameters included phasic volumetric-based LA emptying fractions and LA strain-based indices. LA endocardial and epicardial borders were manually delineated in the two- and four-chamber long-axis images using LV end-diastole as a reference phase. LA appendage and pulmonary veins were excluded from the LA volume. Then, the software automatically traced the atrial border in the subsequent phases. Manual adjustments were performed to obtain optimal tracking of the LA border. The software then computes the LA peak longitudinal reservoir strain (LA-RS), conduit strain (LA-CS), and booster strain (LA-BS). Total LA emptying fraction (LAEFT), a measure of reservoir function, was calculated as (LAV_*max*_–LAV_*min*_)/LAV_*max*_. Passive LA emptying fraction (LAEFP), a measure of conduit function, was calculated as (LAV_*max*_–LAV_*ac*_)/LAV_*max*_. Booster LA emptying fraction (LAEFB), a measure of atrial contractile pump function, was calculated as (LAV_*ac*_–LAV_*min*_)/LAV_*ac*_. Representative images of LV and LA longitudinal strain are shown in Figure 1.

#### Measurement of epicardial adipose tissue volume

**FIGURE 2:**
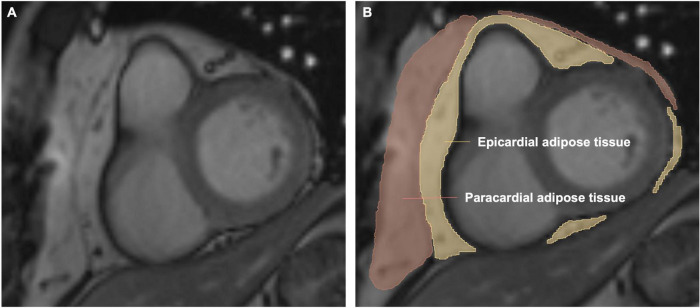
Volumetric assessment of the epicardial adipose tissue (EAT) on short-axis slices. Panels **(A,B)** are representative images of one short-axis slice. Epicardial adipose tissue is shown in yellow. Paracardial adipose tissue is shown in red. Pericardial adipose tissue is epicardial adipose tissue plus paracardial adipose tissue. The EAT volume was calculated by summation of the results of each slice’s area multiplied by the slice thickness based on the modified Simpson’s rule.

The measurement of EAT volume has been previously described (10, 14). The areas of EAT were delineated on consecutive short-axis cine images. Epicardial border and visceral pericardial border on each slice from the level of the mitral valve to the apical slice were manually traced (Figure 2). Then the EAT volume was calculated by summation of the results of each slice’s area multiplied by the slice thickness based on the modified Simpson’s rule.

Morphological LA and LV parameters and the EAT volume were indexed to body surface area (BSA). Reproducibility was assessed for EAT volume and LA and LV strain parameters. To determine the intra-observer reproducibility, one observer (SH) performed all the measurements at first and repeated in 30 randomly selected CMR scans 1 month later. And inter-observer reproducibility was evaluated by comparing the measurements from the same collection of images by another experienced observer (KS).

### Statistical analysis

Statistical analyses were conducted with SPSS (Version 19; IBM, Armonk, NY, USA) and Graphpad Prism (Version 7.0a, GraphPad Software Inc., San Diego, CA, USA). Baseline characteristics and CMR indices were all summarized across the quartile of EAT volume. Continuous data are expressed as the means ± SDs or medians with interquartile ranges. Categorical data are expressed as numbers (percentages). Continuous variables were compared among the four groups of EAT volume using one-way analysis of variance (ANOVA). Dichotomous variables were compared among groups by using χ test. Pearson’s or Spearman correlation analyses were performed to evaluate the bivariable correlations among EAT volume and other CMR parameters as appropriate. Multivariable linear regression analyses were conducted using body mass index (BMI), EAT and clinical factors as independent variables and LA and LV myocardial longitudinal strain as dependent variables. Candidate variables with no collinearity and a *p*-value < 0.1 in the univariate analyses as well as factors based on clinical grounds were included in the multivariable models. The intraclass correlation coefficient (ICC) was used to assess the inter- and intra- observer reproducibility. Two-sided *p* < 0.05 was considered statistically significant.

## Results

### Baseline characteristics of the included participants

In total, 283 postmenopausal women were enrolled in this study. Baseline demographic and clinical characteristics were summarized across the quartiles of EAT volume in Table 1. The ranges of EAT volume of the four groups were Q1: <37.5 ml/m^2^, Q2: 37.5∼48.9 ml/m^2^, Q3: 48.9∼62.0 ml/m^2^, and Q4: > 62.0 ml/m^2^, respectively. The average age of the study cohort was 61.5 ± 9.1 years. Six women in the study population had surgical menopause. There were graded increases in BMI and TG/HDL across the quartiles of EAT. And BMI was significantly correlated with EAT volume (r = 0.354). EAT was inversely correlated with the level of HDL cholesterol (r = −0.214). Age was found to be related to EAT volume (r = 0.399). Women with higher EAT volumes were significantly older than those in the lower quartile. Presence of hypertension was more frequent in participants with a larger EAT volume. The presence of diabetes mellitus had a graded increase from the lowest to the highest quartile of EAT, but the difference was not statistically significant.

**TABLE 1:** Baseline characteristics of the included participants.

Variable	Q1 (*n* = 72)	Q2 (*n* = 71)	Q3 (*n* = 70)	Q4 (*n* = 70)	*P*-value
EAT ranges, ml/m^2^	<37.5	37.5∼48.9	48.9∼62.0	>62.0	–
Age, years	57.3 ± 8.5	59.5 ± 7.4	63.0 ± 9.1	66.4 ± 9.0	**<0.001**
Height, cm	1.57 ± 0.05	1.57 ± 0.05	1.57 ± 0.06	1.56 ± 0.06	0.325
Weight, kg	55.5 ± 7.8	59.4 ± 9.6	60.7 ± 9.9	62.1 ± 8.1	**<0.001**
BSA, m^2^	1.64 ± 0.12	1.69 ± 0.15	1.70 ± 0.15	1.72 ± 0.12	**0.009**
BMI, kg/m^2^	22.4 ± 2.7	24.0 ± 3.3	24.7 ± 3.5	25.6 ± 3.2	**<0.001**
SBP, mmHg	127.9 ± 20.4	132.1 ± 18.8	137.6 ± 17.9	134.0 ± 17.9	**0.045**
DBP, mmHg	79.3 ± 14.3	78.0 ± 11.1	81.7 ± 14.2	78.7 ± 11.4	0.483
Heart rate, min^–1^	79.1 ± 15.5	77.5 ± 13.5	78.1 ± 15.9	77.4 ± 11.7	0.976
Menopausal age, years	50 (48, 51)	49.5 (46.7, 51.2)	50 (48, 50)	49 (47, 50.5)	0.813
Hypertension, *n* (%)	18 (25)	31 (43.7)	41 (58.6)	45 (64.3)	**<0.001**
Diabetes, *n* (%)	22 (30.5)	25 (35.2)	28 (40.0)	31 (44.3)	0.322
Dyslipidemia, *n* (%)	13 (18.1)	20 (28.2)	21 (30.0)	23 (32.8)	0.214
TG, mmol/L	1.32 (0.98, 1.81)	1.29 (0.80, 1.76)	1.50 (1.12, 1.85)	1.58 (1.17, 1.86)	0.055
TC, mmol/L	4.19 (3.45, 4.83)	4.55 (3.82, 5.17)	4.62 (4.03, 5.44)	4.76 (4.21, 5.46)	**0.005**
HDL, mmol/L	1.42 (1.26, 1.54)	1.47 (1.27, 1.71)	1.28 (1.12, 1.51)	1.29 (1.03, 1.53)	0.001
LDL, mmol/L	2.63 (1.96, 3.34)	2.71 (2.21, 3.33)	2.69 (1.85, 3.16)	2.35 (1.83, 2.80)	0.060
eGFR, ml/min/ 1.732 m^2^	89.4 ± 15.5	91.2 ± 16.4	85.6 ± 16.7	79.1 ± 16.2	**0.002**
TG/HDL	0.55 (0.45, 0.97)	0.61 (0.36, 0.97)	0.80 (0.50, 1.15)	0.79 (0.59, 1.28)	**0.005**
EAT volume, ml/m^2^	29.7 ± 5.9	42.7 ± 3.3	54.8 ± 4.1	73.3 ± 9.6	**<0.001**
LV-EDV, ml/m^2^	73.8 ± 10.2	75.1 ± 14.1	74.5 ± 14.1	80.5 ± 23.7	0.756
LV-ESV, ml/m^2^	29.1 ± 6.3	30.6 ± 9.2	29.6 ± 8.1	35.7 ± 21.3	0.825
LVEF,%	61.1 ± 4.9	59.9 ± 6.6	60.3 ± 7.3	57.9 ± 11.0	0.514

The values are the mean ± SD, median (interquartile ranges) and numbers (percentages). EAT, epicardial adipose tissue; BSA, body surface area; BMI, body mass index; SBP, systolic blood pressure; DBP, diastolic blood pressure; TG, plasma triglycerides; TC, total cholesterol; HDL, high-density lipoprotein; LDL, low-density lipoprotein; eGFR, estimated glomerular infiltration rate; LV-EDV, left ventricular end-diastolic volume; LV-ESV, LV end-systolic volume; LVEF, left ventricular ejection fraction. P-values of statistical significance are shown in bold.

### Comparisons of cardiac magnetic resonance-derived cardiac structure and function among quartiles of epicardial adipose tissue volume

**FIGURE 3:**
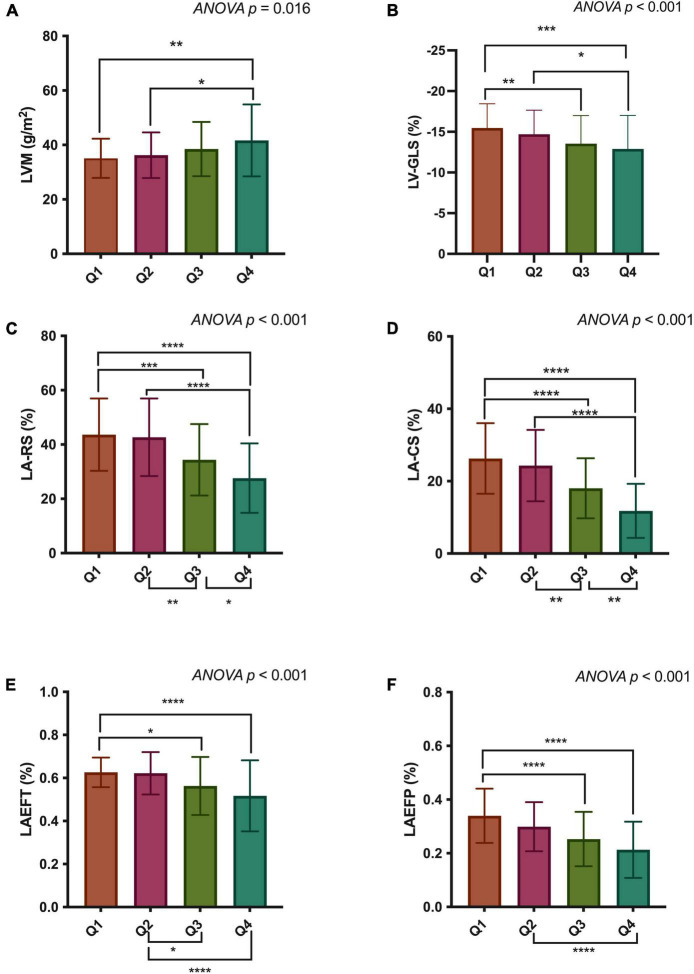
Comparisons of LVM **(A)**, LV-GLS **(B)**, LA-RS **(C)**, LA-CS **(D)**, LAEFT **(E)**, and LAEFP **(F)** among quartiles of epicardial adipose tissue (EAT) volume. LVM, left ventricular mass; LV-GLS, LV global longitudinal strain; LA-RS, left atrial reservoir strain; LA-CS, LA conduit strain; LAEFT, total LA emptying fraction; LAEFP, passive LA emptying fraction. *p < 0.05; **p < 0.01, ***p < 0.001; ****p < 0.0001.

Cardiac structural and functional parameters by CMR according to the quartile distribution of EAT volume are presented in Table 2 and Figure 3. The mean EAT volumes of the four groups were Q1 = 29.7 ± 5.9 ml/m^2^ vs. Q2 = 42.7 ± 3.3 ml/m^2^ vs. Q3 = 54.8 ± 4.1 ml/m^2^ vs. Q4 = 73.3 ± 9.6 ml/m^2^. LVM and LV remodeling index were both increased in the group with the highest EAT volume, compared to those in the lowest quartile. LV-EDV and LV-ESV did not show any difference across the four groups. There was no significant difference in LVEF among the groups. The LV-GLS and LV-GCS, but not LV-GRS, was significantly reduced in the highest quartile of EAT.

**TABLE 2:** Cardiac structural and functional parameters by cardiac magnetic resonance (CMR) and their correlations with epicardial adipose tissue (EAT) volume.

Variable	Q1 (*n* = 72)	Q2 (*n* = 71)	Q3 (*n* = 70)	Q4 (*n* = 70)	Correlation coefficients		
					R	95% CI	*P*-value
LVM, g/m^2^	35.1 ± 7.2	36.2 ± 8.4	38.9 ± 10.5	42.2 ± 13.7	0.204	0.084–0.318	**0.001**
Remodeling index^#^	0.48 ± 0.09	0.49 ± 0.11	0.53 ± 0.11	0.53 ± 0.10	0.242	0.123–0.353	**<0.001**
LV-GRS, %	34.3 ± 8.1	33.3 ± 9.1	32.0 ± 9.7	30.5 ± 10.8	−0.134	−0.257—0.009	**0.031**
LV-GCS, %	−21.0 ± 2.3	−20.6 ± 2.6	−20.0 ± 3.1	−18.9 ± 4.5	−0.174*	−0.307—0.028	**0.005**
LV-GLS, %	−15.5 ± 3.0	−14.7 ± 3.0	−13.5 ± 3.4	−12.9 ± 4.1	−0.250*	−0.379—0.108	**<0.001**
LAV_*max*_, ml/m^2^	32.6 (27.9, 41.2)	37.4 (25.5, 42.3)	37.6 (27.8, 46.9)	38.0 (27.5, 48.2)	0.136	0.006–0.261	**0.035**
LAV_*ac*_, ml/m^2^	20.8 (17.5, 26.8)	25.6 (17.2, 31.6)	27.7 (20.0, 33.7)	28.5 (20.3, 43.6)	0.270	0.145–0.387	**<0.001**
LAV_*min*_, ml/m^2^	12.2 (9.4, 16.7)	13.6 (7.9, 16.6)	14.9 (10.4, 21.2)	16.6 (11.7, 26.7)	0.224	0.097–0.344	**<0.001**
LAEFT, %	0.63 ± 0.07	0.62 ± 0.10	0.55 ± 0.14	0.51 ± 0.17	−0.346	−0.453—0.230	**<0.001**
LAEFP, %	0.34 ± 0.10	0.30 ± 0.09	0.25 ± 0.10	0.21 ± 0.11	−0.442	−0.538—0.334	**<0.001**
LAEFB, %	0.43 ± 0.09	0.46 ± 0.11	0.41 ± 0.15	0.39 ± 0.16	−0.126	−0.248–0.001	0.051
LA-RS, %	43.6 ± 13.3	42.7 ± 14.3	33.9 ± 13.4	27.2 ± 13.0	−0.424	−0.522—0.315	**<0.001**
LA-CS, %	26.2 ± 9.7	24.3 ± 9.9	17.8 ± 8.3	11.6 ± 7.5	−0.527	−0.614—0.426	**<0.001**
LA-BS, %	17.4 ± 5.7	18.7 ± 7.1	16.2 ± 8.2	14.5 ± 7.9	−0.169	−0.289—0.044	0.008

^#^LV remodeling index was calculated as LVM/LV-EDV. *LV-GCS and LV-GLS were calculated as absolute value in the correlation analyses. LVM, LV mass; LV-GRS, LV global radial strain; LV-GCS, LV global circumferential strain; LV-GLS, LV global longitudinal strain; LAV*_max_*, maximum left atrial volume; LAV*_ac_*, pre-atrial contraction LA volume; LAV*_min_*, minimum LA volume; LAEFT, total LA emptying fraction; LAEFP, passive LA emptying fraction; LAEFB, booster LA emptying fraction; LA-RS, LA peak longitudinal reservoir strain; LA-CS, LA peak longitudinal conduit strain; LA-BS, LA peak longitudinal booster strain. P-values of statistical significance are shown in bold.

LAV_*min*_ and LAV_*ac*_ were markedly enlarged in participants with larger EAT volumes. But no significant difference was found in LAV_*max*_ among the groups. Regarding volume-based LA function, LAEFT and LAEFP were markedly decreased in the higher EAT group. No significant difference was observed in LAEFB among the four groups. The strain-based LA function indices, including LA-RS, LA-CS, and LA-BS, all progressively decreased from the lowest quartile of EAT volume to the highest.

### Associations between epicardial adipose tissue and cardiac structural and functional indices

Univariate correlation analyses of EAT and other CMR indices are presented in Table 2. EAT volume had a weak correlation with LVM (r = 0.204) and the remodeling index (r = 0.242). LV-GLS (r = −0.250), LV-GCS (r = −0.174), and LV-GRS (r = −0.134) were inversely correlated with EAT volume. Volume-based LA function indices were negatively related to EAT volume, with only LAEFT (r = −0.346) and LAEFP (r = −0.442) being statistically significant. LA-RS (r = −0.424) and LA-CS (r = −0.527), and LA-BS (r = −0.169) were also correlated with EAT volume.

In the multivariable linear regression analyses, age, systolic blood pressure, heart rate, hypertension, diabetes, dyslipidemia, menopausal age, surgical menopause, TG/HDL, BMI, and EAT volume were included as independent variables. Among them, BMI (β = −0.261), EAT volume (β = −0.149) and diabetes (β = −0.286) were found to be independently associated with LV-GLS. Furthermore, EAT volume was also found to be independently correlated with LA-RS (β = −0.277), LA-CS (β = −0.324), and LA-BS (β = −0.210) (Table 3).

**TABLE 3:** Multivariable linear regression of epicardial adipose tissue (EAT) and cardiac function parameters.

	LV-GLS		LA-RS		LA-CS		LA-BS	
Variables	Univariable β	Multivariable β	Univariable β	Multivariable β	Univariable β	Multivariable β	Univariable β	Multivariable β
Age, years	−0.123*		−0.479*	–0.381	−0.546*	–0.374	−0.191*	–0.213
SBP, mmHg	−0.189*		–0.125		−0.148*		–0.044	
HR, min^–1^	–0.039		0.015		–0.007		0.052	
Hypertension	−0.150*		−0.220*		−0.282*		–0.077	
Diabetes	−0.312*	–0.286	−0.175*	–0.159	−0.178*	–0.166	–0.083	
Dyslipidemia	–0.066		–0.087		–0.109		–0.015	
Menopausal age, years	–0.022		–0.106		–0.099		–0.057	
Surgical menopause	0.056		0.093		0.089		0.065	
TG/HDL	–0.025		–0.081		–0.126		0.004	
BMI, kg/m^2^	−0.243*	–0.261	−0.182*		−0.200*		–0.093	
EAT volume, ml/m^2^	−0.250*	–0.149	−0.424*	–0.277	−0.527*	–0.324	−0.169*	–0.210

Multivariable regression models were constructed with LV and LA myocardial longitudinal strain as dependent variables and age, SBP, HR, hypertension, diabetes, dyslipidemia, menopausal age, surgical menopause, TG/HDL, BMI, and EAT as independent variables. Abbreviations as in Table 2. *p < 0.05 in the univariable linear regression analyses.

### Intra- and inter-observer reproducibility of cardiac magnetic resonance parameters

The intra-observer and inter-observer reproducibility of EAT volume, and LA and LV strains were considered excellent (ICCs ranged from 0.882 to 0.952) (Supplementary Table 1).

## Discussion

In this study, we used CMR to explore the associations of EAT with cardiac functional and structural parameters. First, we found that LV global longitudinal strain was progressively reduced with the increasing of EAT volume. Second, the phasic emptying function and deformation of LA were also found to be gradually decreased from the lowest quartile of EAT volume to the highest. Third, EAT was correlated with LV GLS and LA strains independent of age, systolic blood pressure, heart rate, hypertension, diabetes, dyslipidemia, menopausal age, surgical menopause, TG/HDL, and BMI.

Previous studies on EAT mainly focused on EAT thickness around the right ventricular free wall or pericardial adipose tissue (epicardial adipose tissue plus paracardial adipose tissue) measured by echocardiogram or CT. Kim et al. found that pericardial adipose tissue (PAT) was more strongly associated with the subclinical LV dysfunction than BMI and waist circumference (15). However, EAT is embryologically different from paracardial adipose tissue. And EAT is anatomically more closely connected to the coronary arteries and myocardium than paracardial adipose tissue (16). The proinflammatory cytokines released by EAT can directly impair the myocardium, due to the absence of a fascial plane between the two structures (17). A previous echocardiographic study that included 1,004 participants found that only EAT was significantly correlated with diastolic dysfunction, whereas, PAT was not associated with the decreased diastolic function (18). Therefore, we focused on investigating the specific association of EAT, not PAT, with the cardiac structure and function during the same CMR examination.

### Association of epicardial adipose tissue with volumetric- and strain-based phasic left atrial function

Impairment of LA function has been proposed to precede the development of heart failure in a large longitudinal population of asymptomatic participants (7). In our study, we found that LAEFT and LAEFP, but not LAEFB, gradually decreased as the EAT volume accumulated. Evidence about the booster function of LA is still conflicting (19, 20). Even though the volumetric booster function was not significantly different among the groups, the booster strain along with the other two components of LA strain, demonstrated significant differences across the quartile of EAT in our study. This is because that strain-based indices are more sensitive in evaluating atrial mechanics than volumetric indices (21). As for the reservoir function, it is an indicator for LA compliance, reflecting the relaxation of LA during LV systole. The reservoir strain has been previously demonstrated to be correlated with LV filling pressure (22) and the incidence of heart failure (7). The LA-CS of the conduit phase also had a strong association with EAT volume. Considering that conduit function is mainly influenced by the LV relaxation, the abnormality of conduit strain could indicate an early stage of LV diastolic dysfunction.

In a prior study, the researchers demonstrated that LA strain was correlated with EAT in patients with coexisting obesity and diabetes (23). However, possibly due to the small sample size of this study, no significant correlation was observed between EAT and LA function when the patient group and control group were evaluated separately. The researchers assumed that this might indicate that abnormal LA function only occurs when the EAT volume is over a certain amount. Our study was supportive of this assumption. The volume- and strain-based LA function were all significantly reduced in the highest amount of EAT compared to the low quartile of EAT volume.

### Association of epicardial adipose tissue with left ventricular systolic function

The relationship between EAT and LV diastolic function has been well-established in several echocardiographic studies (24). However, evidence about the association between EAT and LV systolic function remains to be elucidated. Several studies using speckle tracking strain analysis by echocardiography demonstrated that EAT is inversely correlated with LV global longitudinal strain (25, 26). Consistent with these results, we observed that longitudinal strain was reduced in the high EAT group. We assumed that the link between EAT and longitudinal strain could be explained by microvascular dysfunction and interstitial fibrosis induced by adipokines and cytokines secreted by EAT (25). These abnormalities mainly affect the subendocardial layer of the myocardium, leading to a reduction in longitudinal LV mechanics in the subclinical stage of disease.

### Potential mechanisms underlying the influence of epicardial adipose tissue on cardiac structure and function

Several relevant mechanisms have been proposed to explain the associations between EAT and cardiac structure and function (27). The first is that EAT could impair LV diastolic filling by a regional mechanical force. Findings of impaired diastolic function and enlarged LA volume in the absence of LV hypertrophy observed in uncomplicated obesity suggest the mechanical role of local adipose depot around the ventricles (28).

Furthermore, EAT could also affect LV structure and function by a paracrine pathway due to the anatomic proximity of EAT to the myocardium (29). A recent study by Ng et al. which quantified the intramyocardial fat content and myocardial interstitial fibrosis by CMR, suggested that the redundant EAT might impair the contractile function by mediating an increase in myocardial fat accumulation and interstitial fibrosis (25).

Third, in our diverse population of postmenopausal women with a relatively high presence of hypertension, obesity and diabetes, there was a possible systemic inflammatory effect induced by these comorbidities that caused the expansion of EAT volume and abnormalities in the heart (30, 31). Under the influence of systemic inflammation, EAT can release various pro-inflammatory cytokines. Thus, the EAT can further aggravates these systemic inflammatory influences on the myocardium and has a deleterious impact on cardiac structure and function. However, a prior clinical study by Woerden et al. did not observe significant association between EAT and the level of C-reactive protein or leucocytes (32). Possibly because the effect of EAT is too small to be reflected *via* peripheral venepuncture. Also, the sample size is relatively small.

As our correlation analyses showed, age and EAT were both independent factors that inversely correlated with LV-GLS and LA strains. Several previous studies also found a strong relation between age and EAT (14, 33). In the study by de Vos et al. age-adjusted regression analyses showed that EAT was positively related to weight, BMI, waist circumference, waist-to-hip ratio and subclinical coronary atherosclerosis (34). However, we were unable to examine the effect of aging process on EAT expansion and other cardiac abnormalities in this study.

### Limitations

This study has several limitations. First, this was a cross-sectional study. Therefore, we were unable to demonstrate a causal relationship between the increased EAT, laboratory biomarkers, co-morbidities and abnormalities in cardiac structure and function. Second, data on waist circumference, waist to hip ratio or abdominal visceral adipose fat are lacking in the majority of included participants, since this was a retrospective study. Inclusion of these data would have made our study more comprehensive. Third, this study only included postmenopausal women. Whether these results could also be applied to men or premenopausal women needs to be elucidated. Finally, due to the lack of long-term follow-up data, we were unable to evaluate the prognostic role of EAT in our study population. Further longitudinal studies are required to examine the potential of EAT in predicting cardiovascular outcomes.

## Conclusion

The accumulation of EAT is independently associated with LV and LA function in postmenopausal women. These associations support the role of EAT in mediating deleterious effects on cardiac structure and function. The assessment of EAT volume may facilitate clinicians to have added information on the impairment of cardiac function. Researchers could commit themselves to developing medicine targeting the epicardial fat tissue to prevent the cardiac remodeling and dysfunction.

## Data availability statement

The raw data supporting the conclusions of this article will be made available by the authors, without undue reservation.

## Ethics statement

The studies involving human participants were reviewed and approved by West China Hospital of Sichuan University Biomedical Research Ethics Committee. Written informed consent for participation was not required for this study in accordance with the national legislation and the institutional requirements.

## Author contributions

SH and Z-GY designed the study. SH was the major contributor to the manuscript drafting and revisions. LJ and JW were responsible for data collecting and sorting. SH and KS analysed the CMR parameters and interpreted the results. W-FY and W-LQ performed the statistical analyses and prepared the tables and figures. YL and YR helped to revise the manuscript critically for important intellectual content. Z-GY supervised the whole work and revised the manuscript. All authors approved the final manuscript.

## Abbreviations

EAT: epicardial adipose tissue
LV: left ventricular
CMR: cardiac magnetic resonance
LA: left atrial
LVEF: left ventricular ejection fraction
eGFR: estimated glomerular filtration rate
EDV: end-diastolic volume
ESV: end-systolic volume
LVM: LV mass
GRS: global radial strain
GCS: global circumferential strain
GLS: global longitudinal strain
RS: reservoir strain
CS: conduit strain
BS: booster strain
LAV_*max*_: maximum LA volume
LAV_*min*_: minimum LA volume
LAV_*ac*_: pre-atrial contraction LA volume
LAEFT: total LA emptying fraction
LAEFP: passive LA emptying fraction
LAEFB: booster LA emptying fraction
BSA: body surface area
BMI: body mass index
ICC: intraclass correlation coefficient
PAT: pericardial adipose tissue.
