# Supplementary material for: Adverse association of epicardial adipose tissue accumulation with cardiac function and atrioventricular coupling in postmenopausal women assessed by cardiac magnetic resonance imaging
Source: Front Cardiovasc Med. 2022 Nov 11;9:1015983. doi: 10.3389/fcvm.2022.1015983 (PMC9691674; doi:10.3389/fcvm.2022.1015983)
Supplement: Supplementary file 1 [file Table_1.DOCX]

Supplementary appendix

**Table S1 Intra- and Inter-observer reproducibility of EAT and strain parameters**

|  | Intra-observer | |  | Inter-observer | |
| --- | --- | --- | --- | --- | --- |
|  | ICC | 95%CI |  | ICC | 95%CI |
| EAT, ml/m^2^ | 0.918 | 0.863–0.932 |  | 0.882 | 0.832–0.905 |
| LV-GRS, % | 0.912 | 0.862–0.965 |  | 0.887 | 0.768–0.912 |
| LV-GCS, % | 0.915 | 0.879–0.953 |  | 0.898 | 0.801–0.915 |
| LV-GLS, % | 0.933 | 0.898–0.962 |  | 0.902 | 0.811–0.939 |
| LA-RS, % | 0.923 | 0.882–0.943 |  | 0.907 | 0.808–0.923 |
| LA-CS, % | 0.935 | 0.876–0.968 |  | 0.911 | 0.825–0.937 |
| LA-BS, % | 0.921 | 0.832–0.957 |  | 0.899 | 0.772–0.918 |

ICC, interclass coefficient.
